# Factors Associated With Response and Resistance to Cognitive Remediation in Schizophrenia: A Critical Review

**DOI:** 10.3389/fphar.2018.01542

**Published:** 2019-01-10

**Authors:** Stefano Barlati, Giacomo Deste, Alessandro Galluzzo, Anna Paola Perin, Paolo Valsecchi, Cesare Turrina, Antonio Vita

**Affiliations:** ^1^Department of Mental Health and Addiction Services, ASST Spedali Civili, Brescia, Italy; ^2^Department of Clinical and Experimental Sciences, University of Brescia, Brescia, Italy

**Keywords:** schizophrenia, cognitive remediation, predictors, cognitive improvement, functional improvement, treatment personalization

## Abstract

Cognitive impairment is a central feature of schizophrenia and has shown to play a crucial role in the psychosocial function of the disorder. Over the past few years, several cognitive remediation (CR) interventions have been developed for schizophrenia, whose effectiveness has also been widely demonstrated by systematic reviews and meta-analysis studies. Despite these evidences, many questions remain open. In particular, the identification of CR response predictors in patients with schizophrenia is still a topic with equivocal findings and only a few studies have looked for the relationship between CR response or resistance and the biological, socio-demographic, clinical and cognitive features in schizophrenia. The current knowledge on positive or negative response predictors to CR treatment in schizophrenia include: age, duration of illness, premorbid adjustment, baseline cognitive performance, intrinsic motivation, hostility, disorganized symptoms, neurobiological reserve, genetic polymorphisms, the amounts of antipsychotics, the type of CR, etc. The aim of this review is to identify neurobiological, psychopathological, cognitive, and functional predictors of CR response or resistance in schizophrenia, taking into account both cognitive and functional outcome measures. The information obtained could be very useful in planning integrated and personalized interventions, also with a better use of the available resources.

## Introduction

Cognitive impairment is a central feature of schizophrenia (Green et al., 2004; Keefe et al., 2006) and has shown to play a crucial role in the psychosocial function of the disorder (Bowie et al., 2006, 2008). A few years ago, the Measurement and Treatment Research to Improve Cognition in Schizophrenia (MATRICS) initiative identified the presence of seven distinct cognitive domains compromised in schizophrenia (Nuechterlein et al., 2004). Numerous studies have shown that cognitive deficits are one of the main causes of severe functional disabilities associated with schizophrenia and are also related to a worse outcome of the disorder (Green, 1996; Green et al., 2000; Bowie et al., 2006). More in detail, recent findings linked functional outcome to the seven cognitive domains identified by the NIMH's Measurement and Treatment Research to Improve Cognition in Schizophrenia (MATRICS) initiative: verbal learning and memory, visual learning and memory, working memory, speed of processing, reasoning and problem solving, attention, and social cognition (Nuechterlein et al., 2004). Cognitive impairment predicts functional outcome at the same level or even better than positive and negative symptoms and is associated with disability even in phases of clinical remission (Galderisi et al., 2014, 2016). In a comprehensive literature review, Green et al. (2000) highlighted that different cognitive deficits might have an impact on specific areas of psychosocial functioning. Cognitive deficits explain 20–60% of the variance of real-life functioning (Green et al., 2004; Fett et al., 2011). From the greater knowledge of the role of cognitive impairment in schizophrenia, its improvement became an essential goal in the treatment of this disorder (Medalia and Choi, 2009). Despite effectiveness of antipsychotic drug treatment in reducing positive symptoms of schizophrenia, cognitive symptoms have proven to be poorly responsive to such treatments (Nielsen et al., 2015). For this reason, new non-pharmacological interventions to improve cognitive symptoms in schizophrenia are under study, with the ultimate goal to also obtain a better functional outcome (Vita and Barlati, 2018). According to the modern neuroscientific knowledge, the brain would be able of changing and developing throughout lifespan (Kaneko and Keshavan, 2012). In this perspective, CR bases its theoretical principles on the concept of cerebral plasticity and neurogenesis (Eack et al., 2010). Moreover, learning that occurs within a CR intervention, if carried out in a stimulating context, would seem to facilitate the development of the brain plasticity, also improving patient functioning (Bowie et al., 2012). In this context, CR aims to recover cognitive functioning through a series of specific methods and techniques (Barlati et al., 2013). CR strategies can be distinguished into two main models: “compensatory” and “restorative.” The “compensatory” treatments try to eliminate or to bypass the specific cognitive deficit, using the subject's residual cognitive abilities. On the other hand, the “restorative” methods are based on knowledge deriving from neurosciences, in particular neuronal plasticity, and have the objective to correct a specific deficit trying to repair the specific underlying compromised function using the capacity of the brain to develop and repair itself throughout the whole life. Restorative remediation strategies utilize two different approaches: bottom-up or top-down. Bottom-up approaches start with remediation of basic neurocognitive skills, such as attention, and advance to more complex skills, such as problem solving. In contrast, top-down approaches use more complex skills with the aim of improving single and specific neurocognitive domains. Thus, some restorative techniques take into account the use of drill and practice exercises, in order to restore cognitive functions and, possibly, improve neuronal plasticity, while others are based on the implementation of new strategies and tend to favor the generalization in different contexts through the execution of different tasks that involve the use of similar strategies (Barlati et al., 2013).

Over the past few years, several CR interventions have been developed for schizophrenia, whose effectiveness has also been widely demonstrated by systematic reviews and meta-analysis studies (McGurk et al., 2007; Grynszpan et al., 2011; Wykes et al., 2011; Medalia and Saperstein, 2013; Vita et al., 2014). Despite the evidence of CR effectiveness in schizophrenia, many questions remain open. In particular, the identification of CR response predictors in patients with schizophrenia is still a topic with equivocal findings and only a few studies have looked for the relationship between CR response or resistance and the biological, socio-demographic, clinical and cognitive features in schizophrenia (Medalia and Richardson, 2005; Kontis et al., 2013; Medalia and Saperstein, 2013; Bowie et al., 2014). The current knowledge on positive or negative response predictors to CR treatment in schizophrenia include: age, duration of illness, premorbid adjustment, baseline cognitive performance, intrinsic motivation, hostility, disorganized symptoms, neurobiological reserve, genetic polymorphisms, the amounts of antipsychotics, the type of CR, etc.

The aim of this review is to identify neurobiological, psychopathological, cognitive, and functional predictors of CR response or resistance in schizophrenia, taking into account both cognitive and functional outcome measures. The information obtained could be very useful in planning integrated and personalized interventions, with a better use of the available resources.

## Materials and Methods

### Search Strategy

Electronic searches were performed using MEDLINE/PubMed, PsycINFO and EMBASE databases combining the following search terms: “schizophrenia,” “cognitive remediation,” “cognitive rehabilitation,” “cognitive training,” “functional outcome,” “response,” “resistance,” “predictors,” “cognitive improvement,” “functional improvement.” Detailed combinations of the above search terms are available from the authors on request. Two of the authors (SB, GD) independently reviewed the database in order to avoid errors in the selection of articles. In addition, the reference lists of the included articles were carefully hand-searched to further identify other studies of possible interest.

### Selection Criteria

All the studies, meta-analyses, and review articles on cognitive remediation in schizophrenia published until June 2018 have been included. Studies were included according to the following criteria: (a) being an original paper published in a peer-reviewed journal, (b) being an English language paper, and (c) having performed experiments using a CR technique in schizophrenia. Studies on psychological, psychosocial, or psychoeducational interventions only, without any cognitive remediation approach or technique, were not considered.

## Results

### Cognitive and Functional Improvement After CR in Schizophrenia: Preliminary Considerations

First of all, it is crucial to define what does it mean with the improvement and normalization concepts and what the scientific literature affirms about them. A number of studies have computed the minimally important difference (MID) for assessment tools, determining that the discrimination threshold for changes in chronic diseases appears to be approximately half (0.5) a standard deviation (SD) (Norman et al., 2003). Harvey et al. (2006) consider as “improved” those patients with a cognitive amelioration of 0.5 SD and as “normalized” those with an improvement of more than −1 SD. Vita et al. (2013) defined as “improved” those patients with a global cognitive improvement greater than or equal to *Z* = 0.5 and as “not-improved” those with a global cognitive change lower than *Z* = 0.5 from baseline to post-treatment. Furthermore, the same authors defined as “normalized” those patients with a global cognitive change from Z < −0.5 at baseline to Z ≥ −0.5 at post-treatment and as “non-normalized” those with a cognitive change at post-treatment lower than *Z* = −0.5. The definition of Vita et al. is similar, but more restrictive than the previous Harvey's definition.

Overall, scientific literature reports that the rate of improvement and normalization after CR in at least one cognitive domain is around 50 and 40% respectively, but some factors predicted a positive outcome up to 70% in the improvement possibility after CR (Kurtz, 2012). In particular, CR in schizophrenia produces 0.5 SD improvements in measures of cognition and also leads nearly 0.5 SD improvements on measures of function (Kurtz, 2012). In the study performed by our group, 46.2 and 41.8% of patients respectively showed a cognitive and functional improvement and 32.4 and 23.6% respectively achieved a cognitive and functional normalization after CR (Vita et al., 2013). Similar findings are also reported by Medalia and Richardson (2005), showing how 49.5% of patients reached a significant cognitive improvement in at least one cognitive domain after CR (NEAR-Neuropsychological Educational Approach to Remediation). Although with a wider definition of the normalization concept, higher percentages of cognitive normalization have been found by Fiszdon et al. (2005), highlighting that 43% of schizophrenia patients achieved cognitive normalization after CR (NET-Neurocognitive Enhancement Therapy). In the study by Vita et al. (2013), 26 patients received the first 2 subprograms (cognitive differentiation and social perception) of the Integrated Psychological Treatment (IPTcog), and 30 patients received a CACR intervention. The IPT is a group-based structured cognitive behavioral program for schizophrenia in which neurocognitive remediation and social cognitive remediation are integrated with psychosocial rehabilitation (Brenner et al., 1994). The IPT-cog groups, composed of 8–10 patients, attended therapy sessions twice a week, 45 min each session, for 24 weeks. The CACR used the Cogpack (Marker Software®) program. The Cogpack includes different neurocognitive exercises that can be divided into domain specific exercises, aimed at training specific cognitive areas among those known to be impaired in schizophrenia (verbal memory, verbal fluency, psychomotor speed and coordination, executive function, working memory, attention) and non-domain-specific exercises that require the use of various functions at the same time and engage culture, language, and calculation skills. The CACR was administered individually twice a week, in 45-min sessions, for 24 weeks. NEAR approach consists in an individual/group patient (3–10) sessions, integrated with a computer-assisted sessions and noncomputer-assisted sessions. Sessions are of 60 minutes, twice a week (about 4 months) (Medalia et al., 2002). NET approach consists in individual / group sessions, integrated with computer-assisted sessions and noncomputer-assisted sessions. Sessions are of 45 minutes at least 5 times a week (about 6 months) (Bell et al., 2001). In another study, Kurtz et al. (2007) observed that 61% of the participants in the CR condition showed evidence of at least a small (≥0.2 SD) Z score improvement and only 22% showed a large (≥0.8 SD or greater) *Z* score improvement for the working memory domain. In this study, the CR intervention consisted in a 12-month (100 h) standardized computer cognitive exercises designed to improve attention, verbal and non-verbal memory. and language processing through repeated drill-and-practice (Bracy, O. PSS CogRehab, Version 95. Indianapolis, IN: Psychological Software Services, Inc; 1995). In a recent study performed by Bosia et al. (2017), 70% of schizophrenia patients improved in at least one cognitive domain and over 50% obtained a normalized score after CR (Cogpack), consisting in 36 sessions of domain-specific computer-aided exercises, three 1-h sessions a week for 3 months. Presently, one of the challenges facing clinicians and CR developers is a limited understanding of who responds to CR. A number of studies investigated positive and negative response predictors to CR (Choi and Medalia, 2005; Fiszdon et al., 2005, 2006; Medalia and Richardson, 2005; Kurtz et al., 2009; Twamley et al., 2011). These studies found that there are several patient and treatment characteristics, influencing a positive or a negative response to CR. In particular, patient variables include baseline cognitive profile (Fiszdon et al., 2005, 2006; Medalia and Richardson, 2005; Kurtz et al., 2009; Lindenmayer et al., 2017), psychological variables such as motivation (Choi and Medalia, 2005; Twamley et al., 2011) and biological features such as age (Wykes et al., 2009; Kontis et al., 2013), phase of the illness (Bowie et al., 2014), catechol-O-methyltransferase (COMT) polymorphisms (Bosia et al., 2007; Panizzutti et al., 2013), and antipsychotic drugs and genetic polymorphisms (Bosia et al., 2014a). Taken together, these studies identified three broad patient variables that could be useful in tailoring CR: cognitive, psychological, and biological. As for other types of psychosocial interventions, variability in response has been observed among recipients. A better understanding of who is able to benefit from CR would enable clinicians to more effectively refer patients to CR or tailor the intervention to the individual. Finally, treatment variables associated to CR response include the administration methods, such as treatment intensity and frequency, the use of drill and practice and/or strategy learning techniques, the integration of CR with other psychiatric rehabilitation interventions (Medalia and Richardson, 2005; McGurk et al., 2007; Wykes et al., 2011). Table 1 summarizes the most investigated patient and treatment characteristics predicting cognitive and functional response to CR in schizophrenia. Table 2 summarizes the literature main findings about predictive factors influencing CR response in schizophrenia.

**Table 1:** The most investigated predictive factors influencing CR response in schizophrenia.

**Patient predictive factors**
Age
DOI
Phase of illness
Chronicity (number of hospitalizations)
Diagnosis
Premorbid functioning (adjustment)
Pretreatment symptoms severity (positive, negative, disorganized symptoms and hostility)
Pretreatment cognition
Pretreatment functioning
Psychological characteristics (depressed mood, anxiety, cooperative attitude, intrinsic motivation)
Cognitive change after CR and functional outcome
Neurobiological predictive factors (cognitive reserve, genetic variability)
**Treatment predictive factors**
Presence of the therapist
The role of the therapist
Therapeutic alliance
CR characteristics (type of CR treatment)
CACR
Use of strategic CR
Use of drill and practice CR
Use of massed practice
Pharmacological treatment (amounts of antipsychotics intake)
Presence of integrated treatment

*CACR, Computer-Assisted Cognitive Remediation; CR, Cognitive Remediation; DOI, Duration of Illness*.

**Table 2:** Predictive factors influencing CR response in schizophrenia: literature main findings.

**Patient positive response predictors**	**Treatment positive response predictors**
Younger age	Presence of a highly trained therapist
Shorter DOI	The active role of the therapist
Early phase of illness	Therapeutic alliance
Lower pretreatment disorganized symptoms	Use of strategic CR
Lower pretreatment hostility	Use of massed practice
Lower pretreatment negative symptoms	Presence of integrated treatment
Greater intrinsic motivation	Lower amounts of antipsychotics intake at baseline
Greater cognitive improvement after CR	Lower anticholinergic burden at baseline
Greater pretreatment cognitive reserve	
**Patient controversial predictors**	**Treatment controversial predictors**
Chronicity (number of hospitalizations)	Type of CR treatment (CACR vs. non-CACR)
Diagnosis	Use of drill and practice CR
Premorbid functioning (adjustment)	
Cognitive impairment at baseline	
Functional impairment at baseline	
Genetic variability	

*CACR, Computer-Assisted Cognitive Remediation; CR, Cognitive Remediation; DOI, Duration of Illness*.

### Patient Characteristics Predicting Cognitive Response to CR in Schizophrenia

Several studies examined whether patient demographics, illness, or cognitive characteristics are predictive of the amount of change in cognitive performance after CR. Few relationships have been reported consistently.

#### Age and Phase of Illness

With regard to demographics, the impact of age has been of greatest interest. Although in some studies age has been unrelated to cognitive improvement (Fiszdon et al., 2005; Medalia and Richardson, 2005; Wykes et al., 2011) and others have reported mixed results (Thomas et al., 2017), a number of studies confirmed that younger patients are more likely to achieve cognitive improvement after CR, showing that patients over the age of 40 have a lower response to CR than patients under 40 (Wykes et al., 2009; Kontis et al., 2013; Vita et al., 2013; Corbera et al., 2017; Lindenmayer et al., 2017).

There is some evidence that stage of illness—an issue closely related to age—might affect cognitive improvement after CR. A meta-analysis that investigated CR effect in patients at their first psychotic episode (Revell et al., 2015) identified a similar modality in cognitive improvement, but with a lower degree, compared to the results of a previous meta-analysis on chronic patients (Wykes et al., 2011). However, other studies achieved different results. Specifically, in the study by Corbera et al. (2017) the early-stage (25 years or younger; mean duration of illness—DOI = 3.4 years) and early-chronic (26–39 years; mean DOI = 7.6 years) patients receiving CR showed larger improvements in working memory, compared to the late-chronic group (40 years and over; mean DOI = 18.2 years). Furthermore, a study performed by Bowie et al. (2014) demonstrated that early course patients (less than 5 years from the psychotic onset) showed a greater improvement in processing speed and executive functions, compared to chronic patients (more than 15 years of disease) after CR. Authors concluded that DOI was inversely associated with improvement in cognition after a CR intervention. If these results will be confirmed, they could support the full inclusion of CR techniques among those tools to be taken into account in the early intervention programs of schizophrenia (McGurk et al., 2007; Wykes et al., 2009).

For these reasons, there is great interest in determining whether CR interventions make a greater impact on cognitive and functioning outcomes for individuals in the prodromal or early phase of illness and, despite more research is needed in this area, preliminary results seem to be encouraging (Barlati et al., 2012, 2015, 2016; Fisher et al., 2013; Revell et al., 2015; Glenthøj et al., 2017).

#### Illness Characteristics and Psychopathological Status

Studies examining the impact of illness characteristics on the efficacy of CR have focused on diagnosis, chronicity, and symptoms severity. Diagnosis (schizophrenia vs. schizoaffective disorder) and indicators of illness chronicity (number of previous hospitalizations) have not been predictive of treatment response (Medalia and Richardson, 2005; Scheu et al., 2013). Symptoms severity has been found to relate to CR- induced cognitive change in some (Fiszdon et al., 2005; Wykes et al., 2011; Vita et al., 2013) but not all studies (Medalia and Richardson, 2005; Scheu et al., 2013). When a relationship was found, lower baseline symptom severity in conceptual disorganization and hostility (Fiszdon et al., 2005; Vita et al., 2013; Lindenmayer et al., 2017) and lower baseline negative symptoms severity (Lindenmayer et al., 2017) were associated with a greater response to CR. Moreover, CR therapy was more effective when patients were clinically stable (Wykes et al., 2011). Lastly, preliminary data of our group showed a negative correlation between autistic traits in patients with schizophrenia and cognitive change (processing speed, verbal memory, and global cognitive score) after CR (Vita et al., 2018; Abstract presented at Cognition in Schizophrenia 2018: A Satellite Meeting of the Schizophrenia International Research Society. Florence, 4 April 2018).

Other patient characteristics, that have been found to be important in predicting CR efficacy, include some psychological characteristics, such as: anxiety, depression, a cooperative attitude, intrinsic motivation to complete treatment and low self-esteem (Fiszdon et al., 2005; Medalia and Richardson, 2005; Ventura et al., 2014). Poor intrinsic motivation—a central feature of schizophrenia with prevalent negative symptoms—has been associated with a low cognitive performance in patients with schizophrenia and has also been identified as a negative predictor of CR efficacy (Saperstein and Medalia, 2016). In this regard, several structured intervention programs on intrinsic motivation have been investigated, with the aim of optimizing CR effectiveness (Choi and Medalia, 2010; Medalia et al., 2018).

#### Pretreatment Cognitive Profile

Research has also looked at whether baseline cognitive characteristics are predictive of response to CR. Baseline cognitive performance measured by neuropsychological test performance has been found to relate to CR- induced cognitive change in some (Fiszdon et al., 2005; Medalia and Richardson, 2005; Kurtz et al., 2009; Vita et al., 2013) but not all studies (Scheu et al., 2013). Fiszdon et al. (2005) have shown how a better baseline cognitive profile in vigilance and verbal memory, was associated with greater CR efficacy, also predicting a 70% of improvement possibilities in memory tasks (digit span) after NET. Vita et al. (2013) have shown how better baseline performances in executive functions and in verbal memory predicted a greater chance of cognitive normalization after CR. This is in line with previous research findings, in which a good baseline performance in executive functions was associated with a better learning of specific strategies during CR (Velligan et al., 2006). A recent study by Davidson et al. (2016) found that baseline learning potential (LP)—the ability to quickly learn and apply a new skill under testing conditions—significantly predicted skill acquisition in verbal and visuo-spatial memory domains after a 8-week CR intervention in patients with schizophrenia. Furthermore, Benoit et al. (2016) demonstrated that higher initial cognitive insight was significantly correlated with greater improvement in speed of processing and visual memory after CR. Burton and Twamley (2015) found no evidence that unawareness of cognitive impairment is a barrier to participation in or ability to benefit from cognitive training, demonstrating no differences in patients with or without neurocognitive insight in terms of treatment utilization and good treatment outcomes in verbal memory and functional capacity (measured by the UCSD Performance-Based Skills Assessment—UPSA).

Other studies, using a clinician assessment of cognitive functions, have been predictive of treatment response. People with schizophrenia who were rated as less impaired on the Positive and Negative Syndrome Scale (PANSS) Cognitive Factor, an index reflecting difficulties with disorientation, poor attention and abstract thinking, and conceptual disorganization, were found to be more likely to improve after CR (Medalia and Richardson, 2005). Conversely, Pillet et al. (2015) observed numerous negative correlations between cognitive performance at baseline and patients improvements following CR, with a lower cognitive performance predicting greater cognitive improvements. Overall, although better baseline cognitive performance may be associated with greater effectiveness of CR, intervention- induced cognitive improvement is not fully related to baseline neuropsychological performance, and patients can benefit from CR regardless of their level of illness- related cognitive impairment (Twamley et al., 2011; Scheu et al., 2013; Rodewald et al., 2014).

### Patient Characteristics Predicting Functional Response to CR in Schizophrenia

In recent years, a few studies have begun to examine whether patient characteristics at the onset of CR are predictive of post intervention functional change. Age, symptoms, baseline cognitive performance, and pre- intervention functioning have been found to be predictive of change in functional status, including progress in psychosocial rehabilitation programs, community function and role-play measures of social and everyday life skills (Kurtz, 2012).

#### Age and Phase of Illness

Similar to the research on cognitive change, Vita et al. (2013) reported that patient age was predictive of functional improvement (measured by the Global Assessment of Functioning and the Health of the Nation Outcome Scale), with younger patients making greater gains after CR. In line with these findings, Bowie et al. (2014) showed that early course patients had larger improvements in adaptive competence and real-world work skills, than patients in the chronic course of schizophrenia after CR, demonstrating that DOI was inversely associated with improvement in real-world work skills after a CR intervention. Revell et al. (2015) in their meta-analysis on CR in first episode schizophrenia patients identified a similar modality in functional improvement, but with a lower degree, compared to the results of a previous meta-analysis on chronic patients (Wykes et al., 2011). Conversely, other studies reported mixed results on functioning, suggesting that CR may differentially benefit persons with severe mental illnesses depending on age (Thomas et al., 2017).

#### Illness Characteristics and Psychopathological Status

Lower baseline conceptual disorganization and lower positive symptoms severity predicted better social function improvement after CR (Vita et al., 2013; Farreny et al., 2016; Lindenmayer et al., 2017).

#### Pretreatment Cognitive Profile

Baseline attention, working memory and verbal learning ability have been found to be predictive of change in functional capacity after Computer-Assisted Cognitive Remediation (CACR) (Kurtz et al., 2007, 2009), and a trend has been noted for executive functioning to predict post- CR functioning in the community (Vita et al., 2013). In both studies higher baseline performance predicted greater functional gains with CR. Lindenmayer et al. (2017) found a significant association between faster speed of processing, better visual and verbal learning at baseline and greater functional improvement after a systematic cognitive intervention within a rehabilitative setting. Conversely, Bosia et al. (2017) showed no significant effect of baseline cognitive function on functional outcome after CR. This topic is still a matter of debate in literature, as some studies suggested a relation between specific cognitive functions and improvement in psychosocial functioning after CR (Medalia and Richardson, 2005; Kurtz et al., 2009; Vita et al., 2013; Farreny et al., 2016), while others didn't support any relation (Roder et al., 2002; Bosia et al., 2017), or have supported a negative correlation (Twamley et al., 2011; Scheu et al., 2013; Rodewald et al., 2014).

#### Pretreatment Level of Functioning

The term premorbid adjustment refers to a broad set of abilities, including premorbid intelligence quotient (IQ), overall individual's social, interpersonal, academic and occupational functioning prior to the onset of psychotic symptoms (Addington and Addington, 2005). There are few and conflicting data demonstrating a correlation between pre- intervention level of functioning and CR responsiveness. In a study by Bell et al. (2014) participants were assigned to receive either supported employment or supported employment with CR. The lower functioning people with schizophrenia who received supported employment and CR achieved significantly higher employment rates than individuals in supported employment only. In contrast, among higher- functioning participants, the addition of CR to supported employment did not enhance outcomes. Conversely, a recent study by Buonocore et al. (2018a), investigating the effect of premorbid adjustment (measured by the Premorbid Adjustment Scale and proposed as an index of cognitive reserve) on cognitive improvements after CR in schizophrenia patients, confirmed the association between premorbid adjustment and cognitive impairment and the possible role of premorbid adjustment on the capacity to recover from cognitive deficits through CR.

#### Cognitive Change After CR and Functional Outcome

The current findings link specific cognitive change features after CR to changes in psychosocial functioning and several studies in investigating cognitive change after CR found a significant improvement in cognitive and in psychosocial functioning (Fiszdon et al., 2008; Eack et al., 2011; Wykes et al., 2012; Rispaud et al., 2016). In particular, Wykes et al. (2012) emphasized that only one cognitive variable—planning/executive functioning—was a predictor of work improvement, despite moderately sized. Furthermore, Rispaud et al. (2016) provided evidence that not all cognitive domains improvement was the same with respect to functioning improvements. While several cognitive domains improved after CR, only working memory and processing speed improvement predicted a better functioning over 1-year. More in detail, this association was specific to patients with at least a moderate cognitive improvement (0.5 SD) and when authors included patients with a smaller degree of cognitive improvement—small (0.2 SD) or median (0.37 SD)—only an improvement in working memory led to a better functioning. Consistent with these findings, a recent study performed by Bosia et al. (2017) showed that the proportion of normalized cognitive performance–i.e., the number of cognitive domains in which patients achieved a “normal” score after CR with respect to baseline deficits—was the only significant predictor of functional outcome. Overall, together these studies support a relationship between cognitive change after CR and functional outcome, although the importance of improving a single cognitive domain versus the number of domains improved, is still uncertain.

### Neurobiological Factors Predicting Response to CR

Recent international literature identified a number of biological factors that appear to influence CR response. Knowledge of these indicators could have important implications in optimizing and customizing CR intervention, even for patients resistant to standard treatments (Medalia et al., 2018). Among these factors, cognitive reserve and some genetic variables seem to play an important role.

#### Cognitive Reserve

In schizophrenia, there is evidence suggesting that a greater cognitive reserve could modulate and counteract some neurodegenerative processes. Cognitive reserve is a term that describes brain resilience against brain damage. At a neural level, cognitive reserve consists for example in a higher synaptic density, in a greater number of neurons, in the ability to use alternative neural networks or different cognitive strategies (Kaneko and Keshavan, 2012). Thus, pretreatment cognitive reserve—cortical surface area and gray matter volume—has been investigated as a predictor of CR efficacy. In this regard, Keshavan et al. (2011) investigated if cortical pre-treatment brain volume influenced Cognitive Enhancement Therapy (CET) effectiveness on cognition and social cognition in a group of early stages schizophrenia patients. Authors demonstrated that greater basal temporal and frontal cortical surface area and higher gray matter volume broadly predicted a more rapid social cognitive response after CET. These findings were confirmed in a recent study by Penadés et al. (2016) in which a greater basal frontal and temporal cortical thickness was correlated with a higher CR post-treatment improvement in non-verbal memory cognitive domain in schizophrenia patients. Conversely, Kontis et al. (2013) showed a limited impact of cognitive reserve on neurocognitive outcome after CR, highlighting the impact of age on CR outcome.

#### Genetic Variability

COMT gene variability has been associated with cognitive performance in schizophrenia (Diaz-Asper et al., 2008) and with CR response, although results have been equivocal (Bosia et al., 2007, 2014a; Greenwood et al., 2011; Pieramico et al., 2012; Panizzutti et al., 2013; Lindenmayer et al., 2015). In a controlled study on CACR intervention in schizophrenia patients, Bosia et al. (2007) found how COMT polymorphism might predict cognitive gain after 3-months follow-up, with a higher improvement in the Quality of Life Scale (QLS) in Met polymorphism after CR in comparison to Val/Val polymorphism after standard treatment. In line with these findings, a more recent study showed how COMT Val/Met and Met/Met genotype was linked to CR efficacy, with a greater improvement in three MATRICS cognitive domains—verbal learning, visual learning, and attention/vigilance—compared to Val/Val genotype (Lindenmayer et al., 2015). In contrast to these positive findings other studies did not find such a great association (or no association) between COMT Met/Met genotype and greater improvement after CR (Greenwood et al., 2011; Twamley et al., 2014). Moreover, further investigation provided new awareness of the role of COMT in CR response, pointing out a potential interaction between COMT polymorphism and antipsychotic drugs (Bosia et al., 2014a) and serotonin 1A receptor (5-HT1A-R) (Bosia et al., 2014b). Overall, these data suggest how COMT genotype could provide useful information in the selection of an appropriate and personalized pharmacological and rehabilitative treatment in schizophrenia (Medalia et al., 2018).

### Treatment Characteristics Predicting Response to CR

Investigators have examined the impact of treatment factors on response to CR, including CR characteristics and medication.

#### CR Characteristics and the Role of Therapist

Regarding CR treatment characteristics that appear to influence outcome, Medalia and Richardson (2005) found that patients were more likely to respond to CR when a highly trained, doctoral- level therapist provided it, as compared to a clinician with less training. They also found intensity of CR training to be a significant moderator of treatment response. Individuals who completed the training more efficiently, attending at least two sessions a week, benefited significantly more than individuals who attended sporadically. Surprisingly, the literature currently offers very little guidance regarding the amount and intensity of cognitive training needed for CR to be effective. In some clinical trials, intervention “dose” has ranged from 24 to 100 sessions (Fisher et al., 2009; McGurk et al., 2009). In a recent study, Buonocore et al. (2017) investigated whether a longer treatment might further increase CACR efficacy in cognition and functioning. Results supported 3-months CACR efficacy both in cognition and in functioning, suggesting that a longer CR intervention could lead to further advantages in executive functions and daily functioning.

Recently, a panel of experts met to develop recommendations for future studies. They suggested that, at a minimum, programs offer at least 2 h of training weekly for a total of 30 to 40 sessions over a 3-month period (Keefe et al., 2011). Although this guideline is helpful, several CR developers have noted that intervention “dose” is likely to be dependent on treatment goals, with different “doses” needed to produce short- vs. long- term change in cognition or impact other potential treatment targets such as psychosocial functioning or neural systems (Wykes and Spaulding, 2011; Vinogradov et al., 2012). Furthermore, a much larger effect of CR on functioning was found when a strategic approach was adopted (McGurk et al., 2007; Wykes et al., 2011). CR methods based on strategy are very different from those based on training (drill and practice), and these two types of CR techniques almost certainly have different effects on brain activity. In their recent review, Bon and Franck (2018) showed an increase brain activity for the training-based method, but a broader network activation for the strategy-based one. Vita et al. (2013) showed that the type of CR treatment—cognitive subprograms of Integrated Psychological Therapy (IPT-cog) vs. CACR (Cogpack)—was not associated with cognitive gain in schizophrenia patients, but predicted functional improvement (CACR > IPT-cog) in this population.

Moreover, significantly stronger effects on functioning were found when CR is offered as part of broader psychosocial rehabilitation interventions (McGurk et al., 2007; Wykes et al., 2011; Bowie et al., 2012). In their meta-analysis on CR in early schizophrenia, Revell et al. (2015) found that CR's effect on functioning was larger in trials with adjunctive psychiatric rehabilitation and small group interventions. In line with these findings, Buonocore et al. (2018b) showed that a longer standard rehabilitation following CR may lead to a significant and stable (5 years) benefit in terms of daily functioning and QoL in patients with schizophrenia.

In a recent study, Cella and Wykes (2017) found that cognitive improvement after CR was associated with massed practice, number of useful strategies and therapeutic alliance, but improvement in functioning was associated only with therapeutic alliance. Authors highlighted that, as for other psychological therapies, it appears that therapeutic alliance may be an important factor for CR outcomes, particularly functioning, in people with schizophrenia, emphasizing the crucial role of the therapist and his impact on patient motivation. Future research should investigate whether the therapist-patient relationship is a useful variable to be taken into account in choosing a type of CR intervention. In view of a greater treatment personalization, some patients could benefit from a CR intervention mediated by the therapist, others from a computer-based intervention, while others from a home-delivered modality (Medalia et al., 2018).

#### Pharmacological Treatment

In people with schizophrenia, CR is intended to be an adjunct to pharmacotherapy. Few studies have examined the impact of medication- related factors on response to CR. Vita et al. (2013) reported that a lower antipsychotic dose at baseline was the strongest predictor of cognitive and functional improvement after CR. The predictive role of a lower antipsychotic dosage associated with a better cognitive and functional outcome after CR in subjects with schizophrenia may suggest both that patients with a more severe illness could have less benefit from CR, both that high antipsychotic dosages could limit CR effectiveness. Similar findings were obtained from other studies (Rodewald et al., 2014), while others showed the lack of influence of antipsychotic dose on the efficacy of CR (Bosia et al., 2017). A medication- related factor that has been found to influence CR response is anticholinergic burden. Many medications, including a few of the antipsychotics prescribed to treat schizophrenia and some of the medications prescribed to control antipsychotic—related side effects have anticholinergic properties. Vinogradov et al. (2009) examined the level of serum anticholinergic activity at baseline and found that it negatively predicted CR- induced improvement on a global measure of cognition, indicating that this medication factor is adversely impacting response to CR.

## Discussion

CR is a behavioral training technique designed to address cognitive and functional impairments associated with schizophrenia. Metanalytic studies offer a good support for CR efficacy on cognitive and functional outcomes in patients with schizophrenia (McGurk et al., 2007; Wykes et al., 2011). Despite these evidences, many questions remain open. Overall, scientific literature reports that the rate of improvement and normalization after CR in at least one cognitive domain is around 50 and 40%, respectively, but some factors predicted a positive outcome up to 70% in the improvement possibility after CR (Kurtz, 2012). In this perspective, a better understanding of who is able to benefit from CR would enable clinicians to more effectively refer patients to CR or tailor the intervention to the individual (Farreny et al., 2016). In particular, the identification of CR response predictors in patients with schizophrenia is still a topic with equivocal findings and only a few studies have looked for the relationship between CR response or resistance and the biological, socio-demographic, clinical, and cognitive features in schizophrenia. The current knowledge on positive or negative predictors of CR efficacy in schizophrenia include: age, DOI, premorbid adjustment, baseline cognitive performance, intrinsic motivation, hostility, disorganized symptoms, neurobiological reserve, genetic polymorphisms, the amounts of antipsychotics, the type of CR, etc (Choi and Medalia, 2005; Fiszdon et al., 2005, 2006; Medalia and Richardson, 2005; Kurtz et al., 2009; Twamley et al., 2011). These studies found that there are several patient and treatment variables, influencing a positive or a negative response to CR. Research in this field identified three broad patient characteristics probably useful to personalize CR: cognitive, psychological, and biological. In particular, patient variables include baseline cognitive profile (Fiszdon et al., 2005, 2006; Medalia and Richardson, 2005; Kurtz et al., 2009; Lindenmayer et al., 2017), psychological variables such as motivation (Choi and Medalia, 2005; Twamley et al., 2011) and biological variables such as age (Wykes et al., 2009; Kontis et al., 2013), stage of illness (Bowie et al., 2014), COMT polymorphisms (Bosia et al., 2007; Panizzutti et al., 2013), and antipsychotic drugs and genetic interactions (Bosia et al., 2014a). Regarding treatment variables associated to CR response, they include the administration methods, such as treatment intensity and frequency, the use of drill and practice and/or strategy learning techniques and the integration of CR with other psychiatric rehabilitation interventions (Medalia and Richardson, 2005; McGurk et al., 2007; Wykes et al., 2011). Overall, CR seems to be more effective in schizophrenia patients with the following features: younger age, shorter DOI, few disorganized symptoms, greater pretreatment cognitive reserve, greater improvement after CR, and lower dosages antipsychotics in their current treatment. About CR characteristics, a much larger effect of CR on functioning was found when a strategic approach was adopted and when CR was offered as part of broader psychosocial rehabilitation interventions. On the other hand, international scientific literature is controversial on the following predictive factors: genetic variability, cognitive, and functional impairment at baseline (see also Table 2).

Despite these findings, there is a further limit to be taken into account, represented by the numerous correlations and interconnections among the different predictors, which makes very difficult to understand the individual weight of each factor. Furthermore, although CR is described as a single intervention, there are multiple dimensions that may distinguish one approach from another, making it difficult to compare results of one study with another. Currently, one barrier to treatment development is our lack of understanding of the critical elements of the intervention and the relative effectiveness of training techniques and approaches (Saperstein and Kurtz, 2013). Direct comparisons studies of CR techniques, identifying the active components of an intervention approach are needed (Kaneko and Keshavan, 2012). Overall, there are some limits in data interpretation. One meaningful difficulty is that there are many standardized CR programs—computerized and non-computerized, individualized or provided in group sessions—but very few studies compared the efficacy of these interventions with each other. Similarly, many studies didn't quantify cognitive and functional improvements in the same way, making the literature very difficult to compare directly. We have to identify the potential predictors of outcome, allowing us to develop a set of variables for tailoring treatments. Accomplishment of this task will require carefully designed studies that control for potential confounding factors to the study's validity. Currently there are still few data suggesting which type of CR intervention is most effective for a specific patient and there is little rigorous evidence to make decisions on which patient should be excluded from this treatment.

## Conclusions

To date, personalization of CR interventions still depend on a clear case formulation where individual goals are set and an appropriate integrated treatment programme is provided (Wykes, 2018). It is therefore critical to identify clinical, neurobiological and genetic predictors of positive or negative response to CR and future research should identify predictors of CR efficacy and effectiveness, not only at an individual level, but also at a community level for a rational resources allocation (Cella et al., 2015). Moderator analyses should be employed to examine how therapeutic response varies across personal, cognitive and biological factors (Genevsky et al., 2010; Wykes and Spaulding, 2011; Cella et al., 2016). Such studies would require the considerable expansion of the traditional clinical trials framework in psychosocial treatment studies to include neuroimaging and genomics assessments (Eack, 2016). Research findings should then be used to personalize CR intervention, improving its delivery and maximizing its efficacy. The final challenge is to begin transitioning CR from an experimental intervention to one incorporated into standard clinical care and to determine how to make the intervention most effective and accessible for patients and their families.

## Author Contributions

SB, GD, AG, AP, PV, CT, and AV participated in the writing process of the first draft of the manuscript. SB and GD made literature search and independently reviewed electronic databases. SB, GD, and AV revised the final version of the manuscript. All authors contributed to reading and approving the final version of the manuscript.

### Conflict of Interest Statement

The authors declare that the research was conducted in the absence of any commercial or financial relationships that could be construed as a potential conflict of interest.
